# Semaglutide Remodels the Gut Microbiome Beyond Its Appetite‐Suppressing Action: Sex‐Specific Gut‐Brain Axis Implications

**DOI:** 10.1002/cph4.70286

**Published:** 2026-10-21

**Authors:** Suyeun Byun, Morgan R. Sotzen, Madison T. Bento, Mya A. Knappenberger, Mohammed Asker, Justin R. Wright, Regina Lamendella, Chunlong Mu, Karolina P. Skibicka

**Affiliations:** ^1^ Department of Nutritional Sciences Pennsylvania State University State College Pennsylvania USA; ^2^ Department of Physiology and Pharmacology Hotchkiss Brain Institute, Cumming School of Medicine, University of Calgary Calgary Alberta Canada; ^3^ Institute of Neuroscience and Physiology, University of Gothenburg Gothenburg Sweden; ^4^ Wright Labs, LLC Huntingdon Pennsylvania USA; ^5^ Department of Biology Juniata College Huntingdon Pennsylvania USA; ^6^ Department of Microbiology, Immunology and Infectious Diseases, Cumming School of Medicine University of Calgary Calgary Alberta Canada; ^7^ Department of Physiology and Pharmacology Hotchkiss Brain Institute, University of Calgary Calgary Alberta Canada

**Keywords:** glucagon‐like peptide 1, gut microbiome, gut‐brain axis, interorgan communication, pair‐feeding, semaglutide, sex differences

## Abstract

The gut microbiome communicates bidirectionally with the brain and peripheral metabolic organs to regulate appetite and body weight. Semaglutide (SEMA), a GLP‐1 receptor agonist, produces robust weight loss by reducing food intake, largely through brain GLP‐1 receptors, yet whether its effects on the gut microbiome are secondary to this centrally driven hypophagia is poorly understood. Given the profound impact of SEMA on food intake and body weight, we propose that SEMA treatment reshapes gut microbial composition and that these shifts are correlated with metabolic outcomes. Diet‐induced obese rats received a 4‐week SEMA treatment and gut microbial composition was analyzed using 16S rRNA gene sequencing. To differentiate the impact of reduced food intake associated with SEMA from other effects of the drug treatment on the gut microbiome, a pair‐fed to SEMA group was tested. SEMA significantly reduced body weight gain and food intake in both sexes. Significant SEMA‐induced changes in microbial richness and evenness were detected. Linear discriminant analysis Effect Size (LEfSe) identified enrichment of Firmicutes taxa—particularly members of the Clostridiaceae family—in SEMA‐treated rats, whereas controls showed greater representation of Bacteroidota taxa. Sex‐specific associations between host metabolic parameters and gut microbial composition were also identified. These findings demonstrate profound, not necessarily health positive, partly sex‐specific gut microbial community shifts following GLP‐1 analogue treatment. Many of the changes in the gut microbiota were not simply driven by the decreased food intake. Thus, GLP‐1 analogue treatment engages the gut microbiome through both food intake–dependent (brain‐to‐gut) and intake‐independent pathways.

## Graphical abstract

Results demonstrate profound, not necessarily health positive, partly sex‐specific, gut microbial community shifts following GLP‐1 analogue treatment. Many of the changes in the gut microbiota were not simply driven by the decreased food intake and weight loss associated with the drug treatment.

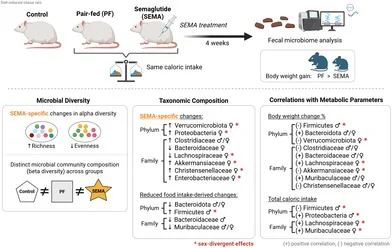

## 1 Introduction

Pharmacological approaches to obesity have advanced rapidly, with glucagon‐like peptide‐1 (GLP‐1) receptor agonists (GLP‐1RAs) emerging as highly effective agents for inducing significant weight loss and improving metabolic health (Wilding et al. 2021; Drucker 2018). Semaglutide (SEMA), a long‐acting GLP‐1 analogue, produces substantial reductions in body weight primarily through decreased food intake, alongside effects on gastrointestinal motility and nutrient handling (Wilding et al. 2021; Holst 2007). While these mechanisms are well established, their downstream consequences for other physiological systems, including the gut microbiome, remain less clearly defined.

The gut microbiome is a key regulator of host metabolism, contributing to energy balance, nutrient processing, and signaling pathways that influence metabolic homeostasis (Turnbaugh et al. 2006; Lynch and Pedersen 2016). Beyond local effects in the gut, the microbiome functions as a signaling organ in interorgan communication: microbial metabolites such as short‐chain fatty acids and secondary bile acids, together with microbiota‐driven release of enteroendocrine hormones including GLP‐1 and peptide YY, reach the brain through vagal afferents and the circulation to influence appetite and body weight, and also act on adipose tissue and liver (De Vadder et al. 2014; Koh et al. 2016; Tolhurst et al. 2012). Conversely, the brain shapes the gut microbial environment through its control of food intake, diet selection, and autonomic regulation of gut motility and secretion (Cryan et al. 2019; David et al. 2014; Mayer et al. 2015). Although early work suggested relatively simple associations between obesity and microbial composition, particularly shifts in the relative abundance of major phyla such as Firmicutes and Bacteroidota (Turnbaugh et al. 2006; Ley et al. 2006), subsequent studies have demonstrated that microbiome–metabolism relationships are highly variable and context‐dependent, resisting reduction to single taxonomic signatures (Sze and Schloss 2016). This complexity raises an important question in the context of pharmacological weight loss: whether interventions such as GLP‐1RAs drive predictable microbiome shifts based on weight loss and hypophagia, or instead produce distinct microbial configurations shaped by drug‐specific physiological effects.

Emerging evidence indicates that GLP‐1RAs can influence gut microbial composition, although the extent and consistency of these effects remain unclear. In rodent models, liraglutide treatment has been shown to alter microbial community structure and diversity, supporting a potential role for GLP‐1 signaling in shaping the gut microbiome (Zhang et al. 2018; Charpentier et al. 2021). In contrast, clinical data are more variable; randomized controlled studies in individuals with Type 2 diabetes report modest or inconsistent microbiome changes following GLP‐1RA treatment, with substantial inter‐individual variability (Smits et al. 2021; Aron‐Wisnewsky et al. 2021). More recent literature suggests that interactions between GLP‐1 signaling and the gut microbiome are complex and bidirectional, involving microbial regulation of GLP‐1 secretion as well as drug‐induced changes in microbial ecology (Zeng et al. 2024; Ganamurali and Sabarathinam 2026). Importantly, these reviews highlight that current evidence remains limited and heterogeneous, with significant gaps in understanding the causal role of GLP‐1RAs in microbiome modulation (Johnson et al. 2026). A major limitation of the existing literature is the difficulty of disentangling direct pharmacological effects of the treatment from those driven by reduced caloric intake and weight loss. Because GLP‐1RAs robustly suppress food intake and alter gastrointestinal physiology, microbiome changes observed during treatment may reflect reduced nutrient availability or altered intestinal transit, making it difficult to distinguish these indirect effects from direct drug‐specific mechanisms. Indeed, caloric restriction alone is sufficient to induce substantial and functionally relevant remodeling of the gut microbiome (David et al. 2014; Fabbiano et al. 2018; Thaiss et al. 2016), highlighting the importance of isolating drug‐specific effects through experimental designs that explicitly control for energy intake.

Biological sex represents an important but underexplored factor in shaping host–microbiome interactions. Sex differences in microbial composition and host physiology are well documented (Kim et al. 2020; Jašarević et al. 2016), yet their role in modulating microbiome responses to pharmacological weight loss has received limited attention. Given that GLP‐1 signaling engages pathways that exhibit sexual dimorphism (Börchers and Skibicka 2025; Richard et al. 2016; Lopez‐Ferreras et al. 2023; López‐Ferreras et al. 2019, 2018; Vogel et al. 2016), it is plausible that microbiome responses to GLP‐1RAs are likewise sex‐dependent. Consistent with this, our recent study in diet‐induced obese rats suggests that metabolic and physiological responses to SEMA differ between males and females (Byun et al. 2025), further supporting the need to consider sex as a biological variable in microbiome studies.

Because SEMA suppresses food intake largely through GLP‐1 receptors in the brain (Gabery et al. 2020), the microbiome consequences of treatment can arise through a brain‐to‐gut route, in which centrally driven hypophagia and altered diet choice change the substrates reaching the microbiota, or through intake‐independent actions of the drug. Here, we investigated the effects of chronic SEMA on gut microbiome composition in diet‐induced obese male and female rats using a controlled experimental design that included a pair‐fed group matched for SEMA caloric intake and diet choice. This approach enabled us to distinguish microbiome changes associated with SEMA treatment from those driven by reduced food intake. We characterized microbial diversity, taxonomic composition, and predicted functional pathways, and examined their relationships with host metabolic parameters. Together, this design allowed us to ask how a centrally and peripherally acting anti‐obesity drug communicates with the gut microbial ecosystem, and whether the resulting microbial changes relate to whole‐body metabolic outcomes.

## 2 Materials and Methods

### 2.1 Animals

Male and female Sprague–Dawley rats (*n* = 36 per sex; 7 weeks old at arrival; Charles River Laboratories) were housed individually under controlled temperature and a 12‐h light/dark cycle. Following a one‐week acclimation period with free access to standard chow (PicoLab Rodent Diet 5053) and water, rats were transitioned to a diet‐induced obesity model by providing a 60% high‐fat diet (HFD; Research Diets, D12462) for 6 weeks prior to treatment initiation and throughout the experimental period, for a total of 10 weeks of obesogenic diet exposure. Fiber consumption was calculated based on daily food intake and the dietary fiber content of each diet (50 mg/g for HFD and 44 mg/g for standard chow), as specified by the manufacturers. All experimental procedures were approved by the Pennsylvania State University Institutional Animal Care and Use Committee.

### 2.2 Drugs

Semaglutide (TLC Pharmaceutical Standards, USA) was prepared in sterile water and administered subcutaneously using a stepwise dose‐escalation protocol to minimize adverse effects. The dose was gradually increased from 7 to 15, 30, and 50 μg/kg, with each dose administered for two consecutive days before escalation to the next dose. Following the 50 μg/kg dose, semaglutide was increased to the target dose of 70 μg/kg, which was then maintained for the remainder of the study, as we and others previously described (Byun et al. 2025; Cawthon et al. 2023). Injection volumes were adjusted based on body weight (1 mL/kg), and control animals received equivalent volumes of vehicle.

### 2.3 Pair‐Feeding Design

To isolate drug‐specific effects from reduced caloric intake, a pair‐feeding design was implemented. For each sex, rats were assigned to control, SEMA‐treated, or pair‐fed (PF) groups, with group assignment performed to balance baseline body weight across groups. PF animals were yoked to SEMA‐treated counterparts and received the same amount and composition of food consumed by their matched partner on the previous day. While control and SEMA‐treated animals had free access to both chow and high‐fat diet, PF animals were provided fixed quantities of each diet to match intake.

### 2.4 Fecal Microbiome Analysis

Fecal pellets were collected following 4 weeks of treatment and submitted to the Wright Laboratory (Huntingdon, PA, USA) for bacterial community profiling using 16S rRNA gene sequencing. Genomic DNA was extracted using the ZymoBIOMICS DNA/RNA Miniprep Kit (Zymo Research), and DNA concentrations were quantified using a Qubit 4 Fluorometer with the dsDNA High Sensitivity Assay Kit (Thermo Fisher Scientific). Amplicon libraries were generated following standard protocols, pooled at equimolar concentrations, purified using SPRI beads, and quality assessed using both Qubit and Agilent BioAnalyzer systems prior to sequencing. Libraries were sequenced on an Illumina MiSeq platform using 2 × 250 bp paired‐end chemistry. Raw sequence data were processed using QIIME2. Reads were quality filtered, truncated (forward: 249 bp; reverse: 166 bp), and denoised using the DADA2 pipeline to generate amplicon sequence variants (ASVs). Taxonomic classification was performed using a Naive Bayes classifier trained on the SILVA 138 database (515F/806R region). Non‐bacterial sequences, including mitochondrial and chloroplast reads, were removed prior to downstream analyses.

Predictive functional profiling was performed using PICRUSt2 implemented within QIIME2 to infer the functional capabilities of the microbial communities. Predicted Kyoto Encyclopedia of Genes and Genomes (KEGG) orthologs were grouped into KEGG pathways using a custom Python script, and pathway abundance data were normalized to counts per million (CPM) prior to downstream analyses. Linear discriminant analysis Effect Size (LEfSe) was used to identify differentially abundant predicted functional pathways between experimental groups using the following criteria: Kruskal–Wallis test *p* < 0.05 and logarithmic LDA score > 2.

### 2.5 Statistical Analysis

Data are presented as mean ± SEM. Body weight, food intake, and other continuous variables were analyzed using one‐way ANOVA with treatment (control, PF, SEMA) as the factor, followed by Holm‐Sidak post hoc tests where appropriate (GraphPad Prism 10). Alpha diversity metrics (observed richness, Pielou's evenness, and Shannon index) were calculated following rarefaction and compared using the Kruskal–Wallis test followed by Dunn's multiple comparisons. Beta diversity was assessed using weighted UniFrac distances and visualized via principal coordinates analysis (PCoA), with group differences evaluated using PERMANOVA with FDR correction. Differentially abundant taxa were identified using LEfSe (LDA > 2.0, *p* < 0.05) following summary of the ASV table at the species (L7) taxonomic rank. Pearson correlation analysis was used to assess relationships between host metabolic parameters and microbial abundance. Heatmaps were generated using z‐score normalized values for visualization purposes. Statistical significance was set at *p* < 0.05.

## 3 Results

### 3.1 Effects of Semaglutide and Pair‐Feeding on Body Weight and Food Intake

In males, baseline body weight did not differ among groups (data not shown). Cumulative food intake (kcal; accumulated over the 2 days of each dose administration during the escalation period) was not altered by the administration of the lowest dose (7 μg/kg) of SEMA (Figure 1B), but each subsequent dose significantly reduced food intake (*p* < 0.001—*p* < 0.0001; Figure 1B). Maintenance dose (70 μg/kg) reduced food intake as expected over the 3 weeks of its administration in both groups (*p* < 0.001; Figure 1C), as we previously reported in Byun et al. (2025). Fiber consumption (mg) was significantly reduced in the PF and SEMA groups compared with controls by all doses of SEMA during escalation (*p* < 0.05—*p* < 0.0001; Figure 1F) as well as maintenance period (*p* < 0.001; Figure 1G). Surprisingly, the proportion of calories derived from HFD was significantly higher in the SEMA group and its corresponding PF group compared with controls during escalation (*p* < 0.01—*p* < 0.0001; Figure 1J) and maintenance periods (*p* < 0.001; Figure 1K). Cumulative body weight change (%) was not reduced by the lowest dose but was markedly reduced by the second SEMA dose and all higher doses compared to control (*p* < 0.001—*p* < 0.0001) and equivalent pair‐feeding rats (*p* < 0.0001; Figure 1N). Moreover, during the 3‐week maintenance period SEMA‐treated rats lost more weight than PF rats (*p* < 0.05; Figure 1O).

**FIGURE 1:**
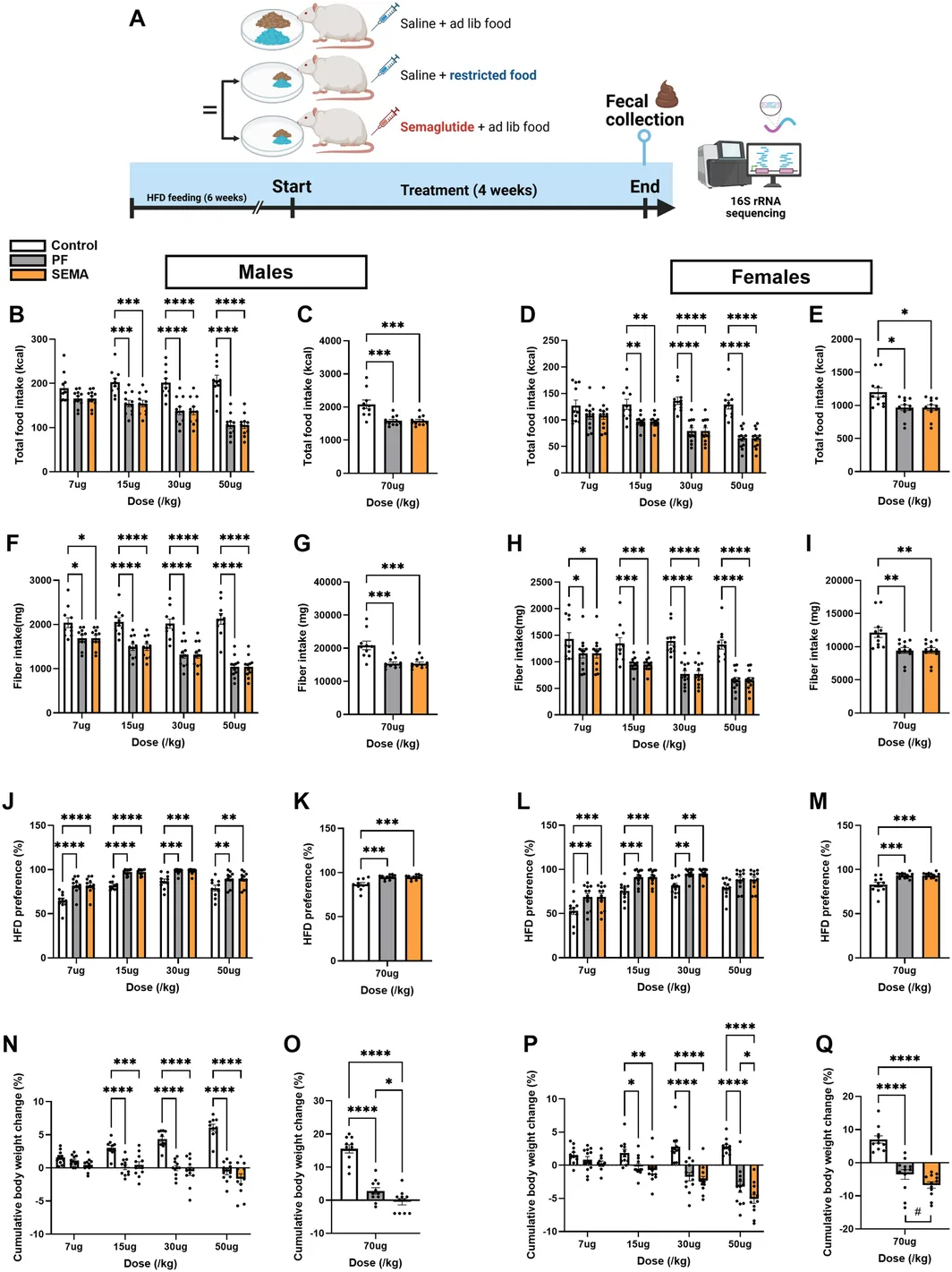
Effects of semaglutide treatment and pair‐feeding on body weight and food intake in male and female rats. An overview of the experimental timeline and study design (A). Cumulative food intake during the semaglutide (SEMA) dose escalation period (B) and maintenance (C) in males and females (D, E). Total fiber consumed (mg) across the treatment period in males (F, G) and females (H, I), respectively. Proportion of caloric intake derived from high‐fat diet (HFD), expressed as percentage of total caloric intake in males (J, K) and females (L, M). Despite reduced total caloric intake, pair‐fed (PF) and SEMA rats consumed a greater proportion of calories from HFD compared with controls at all phases of the treatment. Cumulative body weight change (%) during the escalation and 3‐week SEMA maintenance period in males (N, O) and females (P, Q). Data are presented as mean ± SEM. *p < 0.05, **p < 0.01, ***p < 0.001, ****p < 0.0001. *n* = 10–11/group (males); *n* = 11–12/group (females).

In females, baseline body weight was comparable among groups (data not shown). Cumulative food intake (kcal) was not altered by the administration of the lowest dose of SEMA (7 μg/kg) in females as well (Figure 1D), but each subsequent dose significantly reduced food intake (*p* < 0.01–*p* < 0.0001; Figure 1D). Maintenance dose reduced food intake as expected over the 3 weeks of its administration in both groups (*p* < 0.05; Figure 1E), as we previously reported in Byun et al. (2025). Fiber consumption (mg) was significantly reduced in the PF and SEMA groups compared with controls by all doses of SEMA during escalation (*p* < 0.05–*p* < 0.0001; Figure 1H) as well as maintenance period (*p* < 0.01; Figure 1I). Surprisingly, the proportion of calories derived from HFD was significantly higher in the SEMA group and its corresponding PF group compared with controls during escalation (*p* < 0.01–*p* < 0.001; Figure 1L) and maintenance periods (*p* < 0.001; Figure 1M). Cumulative body weight change (%) was not reduced by the lowest dose but was markedly reduced by the second SEMA dose and all higher doses compared to control (*p* < 0.01–*p* < 0.0001) and equivalent pair‐feeding rats (*p* < 0.05–*p* < 0.0001; Figure 1P). During the highest dose maintenance period, body weight tended to be lower in SEMA‐treated rats than in PF rats (*p* = 0.069; Figure 1Q).

### 3.2 SEMA Has a Unique Impact on Alpha Diversity, Partly Independent From Caloric Restriction

To examine treatment‐associated changes in within‐sample microbial diversity, alpha diversity was assessed using observed species richness, Pielou's evenness, and the Shannon diversity index.

In males, microbial richness differed significantly across groups, with SEMA treatment increasing the number of observed species compared with control rats (*p* < 0.01) (Figure 2A). In contrast, Pielou's evenness was reduced in SEMA rats compared with controls (*p* < 0.05) (Figure 2B), suggesting a shift toward dominance of specific taxa. Shannon diversity did not differ between groups (Figure 2C).

**FIGURE 2:**
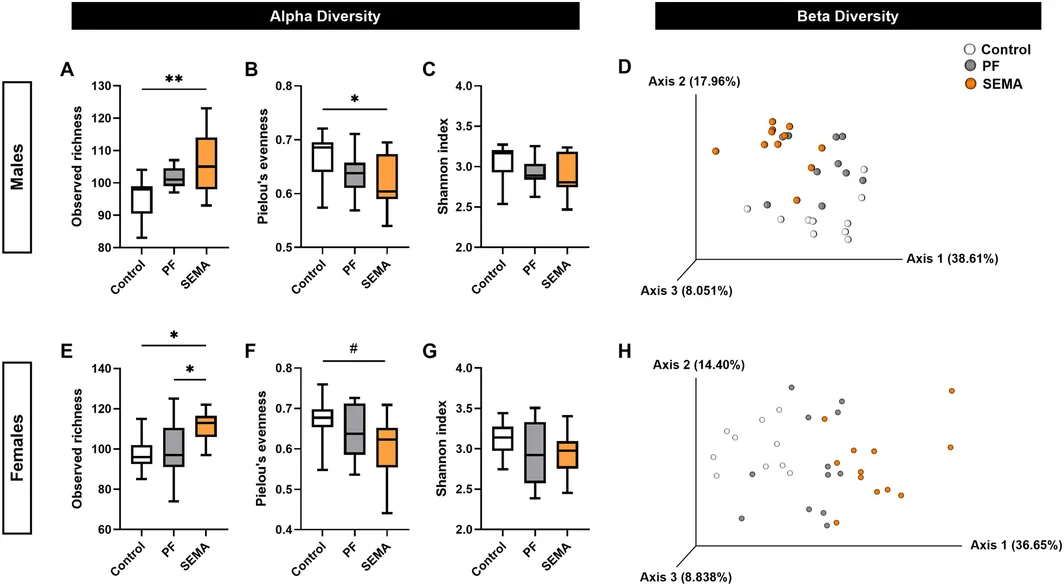
Semaglutide alters gut microbial diversity and community structure. Alpha diversity metrics for male rats (A–C), including observed species richness (A), Pielou's evenness (B), and Shannon index (C). Semaglutide (SEMA) treatment increased microbial richness compared with controls, whereas Pielou's evenness was reduced in SEMA‐treated males. Shannon diversity did not differ among groups. Alpha diversity metrics for female rats (E–G), including observed species richness (E), Pielou's evenness (F), and Shannon index (G). SEMA treatment increased microbial richness compared with control and PF groups. Pielou's evenness showed a trend toward a reduction in the SEMA group compared with controls, although this did not reach statistical significance. Shannon diversity did not differ among groups. Principal coordinates analysis (PCoA) of weighted UniFrac distances illustrating beta diversity in male (D) and female (H) rats. The first three axes explain 38.61%, 17.96%, and 8.05% of the variance in males, and 36.65%, 14.40%, and 8.84% in females. Separation of samples across treatment groups indicates treatment‐associated shifts in overall microbial community composition. Alpha diversity differences were evaluated using the Kruskal–Wallis test followed by Dunn's multiple comparisons, and beta diversity differences were assessed using PERMANOVA with FDR correction. Data are presented as box plots showing median and interquartile range. *n* = 10–11/group (males); *n* = 11–12/group (females). #*p* < 0.1, *p < 0.05, **p < 0.01.

In females, microbial richness was also increased in the SEMA group compared with both control (*p* < 0.05) and PF rats (*p* < 0.05) (Figure 2E). Pielou's evenness did not reach statistical significance but showed a strong trend toward reduced evenness in SEMA rats compared with controls (*p* = 0.06) (Figure 2F). Consistent with this, Shannon diversity did not differ between groups (Figure 2G).

### 3.3 SEMA and Caloric Restriction Induce Significant and Partly Unique Changes in Beta Diversity

To assess treatment‐associated differences in overall microbial community composition, beta diversity was calculated.

In males, the first three axes explained 38.61%, 17.96%, and 8.05% of the total variance, respectively (Figure 2D). PERMANOVA with FDR correction revealed significant differences in microbial community composition among all treatment groups, including control versus PF (*q* < 0.05), control versus SEMA (*q* < 0.01), and PF versus SEMA (*q* < 0.05), indicating that both SEMA and PF groups altered microbial community structure relative to controls, but did so in a partly divergent manner.

Similarly, in females, PCoA of weighted UniFrac distances showed that the first three axes explained 36.65%, 14.40%, and 8.84% of the variance (Figure 2H). PERMANOVA analysis also revealed significant differences in beta diversity across all pairwise group comparisons, including control versus PF (*q* < 0.01), control versus SEMA (*q* < 0.01), and PF versus SEMA (*q* < 0.01), indicating distinct microbial community compositions among all treatment groups in females.

### 3.4 Treatment and Sex‐Specific Shifts of the Taxonomic Composition at Phylum and Family Levels

To further characterize treatment‐associated shifts in gut microbial composition, the relative abundance of bacterial taxa was examined at the phylum and family levels. The analysis focused on the top five most abundant phyla and the top 15 most abundant families across samples.

At the phylum level, the gut microbiota of both male and female rats was dominated by Firmicutes across all treatment groups (Figure 3A,C). In males, the relative abundance of Firmicutes increased in PF and SEMA groups compared with controls, accompanied by a reduction in Bacteroidota (Figure 3A; 63% reduction in PF and 76% reduction in SEMA group). Relative abundance of Verrucomicrobiota was reduced in PF (by 63%), but not SEMA‐treated male rats. In females, Firmicutes remained the dominant phylum across groups (Figure 3C), while Bacteroidota was reduced in SEMA‐treated and PF rats (49% reduction in PF, and 66% in SEMA rats), together with a relative increase in Verrucomicrobiota (100% increase in PF, 155% increase in SEMA, compared to controls). Thus, sex divergent shifts in Verrucomicrobiota were identified.

**FIGURE 3:**
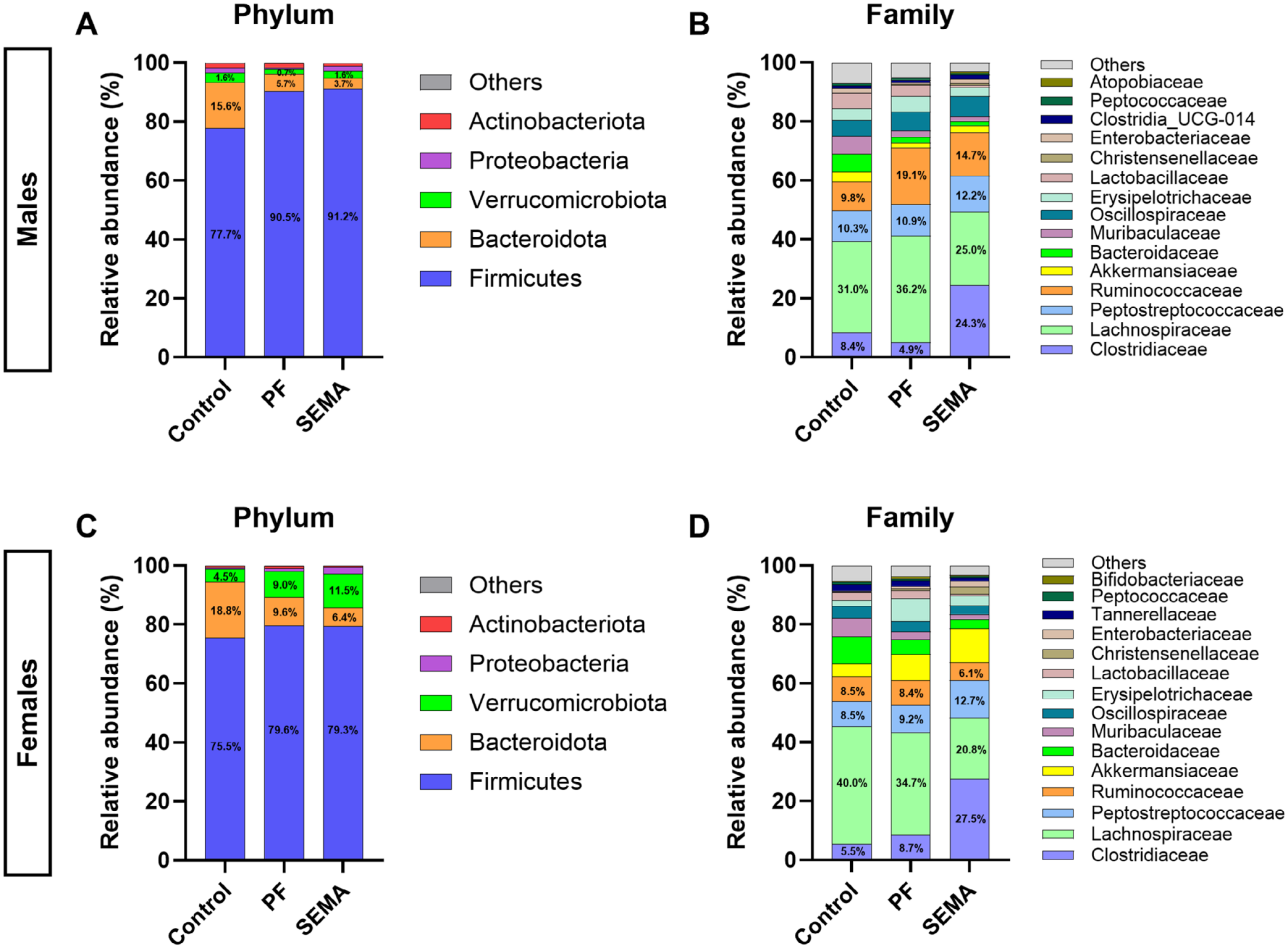
Relative abundance of the top bacterial taxa across treatment groups. Relative abundance of the top five bacterial phyla in male (A) and female (C) rats across treatment groups. The gut microbiota was primarily dominated by Firmicutes, with additional contributions from Bacteroidota, Verrucomicrobiota, Proteobacteria, and Actinobacteriota. Taxa outside the top five most abundant phyla are grouped as “Others.” Relative abundance of the top 15 bacterial families in male (B) and female (D) rats. Dominant families across groups include Clostridiaceae, Lachnospiraceae, Peptostreptococcaceae, and Ruminococcaceae, while additional families such as Akkermansiaceae, Bacteroidaceae, and Muribaculaceae show treatment‐associated differences in relative abundance. Taxa outside the top 15 most abundant families are grouped as “Others.”

At the family level, the top 15 most abundant bacterial families were visualized to highlight treatment‐associated compositional differences (Figure 3B,D). In males, dominant (the top four) families included Clostridiaceae, Lachnospiraceae, Peptostreptococcaceae, and Ruminococcaceae. Notably, Clostridiaceae was enriched (3‐fold increase, unique to SEMA and not PF) whereas the relative abundance of Lachnospiraceae was uniquely reduced in the SEMA group compared with control and PF rats (19% reduction compared to control and 30% reduction compared to PF), while Bacteroidaceae and Muribaculaceae were relatively more abundant in controls (Bacteroidaceae: 68% reduction in PF and 75% reduction in SEMA; Muribaculaceae: 62% reduction in PF and 74% reduction in SEMA). In contrast, Ruminococcaceae was elevated in PF rats (2‐fold increase) and SEMA rats (50% increase) (Figure 3B).

In females, several family‐level shifts were also observed (Figure 3D). Clostridiaceae (5‐fold increase), Peptostreptococcaceae (1.5‐fold increase), and Akkermansiaceae (2.5‐fold increase) appeared more prominent in SEMA‐treated rats, whereas Lachnospiraceae, Bacteroidaceae, and Muribaculaceae were relatively reduced compared with control and PF groups (48%, 64%, 73% reductions, respectively). In contrast to results in males, Ruminococcaceae were not altered by the treatment. Also, the increase in Akkermansiaceae was unique to females and most potent in the SEMA‐treated group.

Overall, these patterns suggest that SEMA treatment is associated with shifts in the relative abundance of major bacterial phyla and restructuring of dominant bacterial families within the gut microbiome, in some cases unique to SEMA and in others driven by caloric restriction. Sex‐specific shifts were also detected.

### 3.5 Largely Unique to SEMA Effects on Taxa Counts

To further investigate treatment‐associated microbial shifts at a finer taxonomic level, the abundance of selected genera was compared across treatment groups. Sequencing depth did not differ across groups, as two‐way ANOVA revealed no significant effects of treatment or sex, and no interaction (data not shown), indicating comparable sampling across conditions.

Within the Firmicutes lineage, SEMA treatment, but not PF, was associated with increased abundance of several taxa in both sexes, including *Clostridium sensu stricto 1*, *Clostridium perfringens* , and *Terrisporobacter* (Figure 4A–C). These were enriched in SEMA‐treated rats compared with both control and PF groups. The Christensenellaceae R‐7 group increased following SEMA treatment, but only in females (Figure 4D). Similarly, the NK4A214 group (Oscillospiraceae family) showed a marked increase in SEMA‐treated females (Figure 4E). Within the Lachnospiraceae family, distinct patterns were observed across taxa. The genera *Blautia* and *ASF356* showed decreased abundance following SEMA treatment compared with control and PF groups, with the reduction in *ASF356* observed predominantly in females (Figure 4F–G). In contrast, the genus *Sellimonas* displayed higher abundance in both PF and SEMA groups relative to controls in females (Figure 4H), suggesting that this shift may reflect caloric restriction‐associated changes rather than a SEMA‐specific effect. Interestingly, they were reduced by caloric restriction alone, but not in the SEMA‐treated males. Within the Bacteroidota lineage, both *Bacteroides* and *Muribaculaceae* showed reduced abundance in SEMA‐treated rats across both sexes (Figure 4I,J), and these changes appeared to be driven by caloric restriction. Finally, within the Proteobacteria phylum, the genus *Escherichia‐Shigella* was markedly enriched in SEMA‐treated rats, with a stronger increase observed in females (Figure 4K).

**FIGURE 4:**
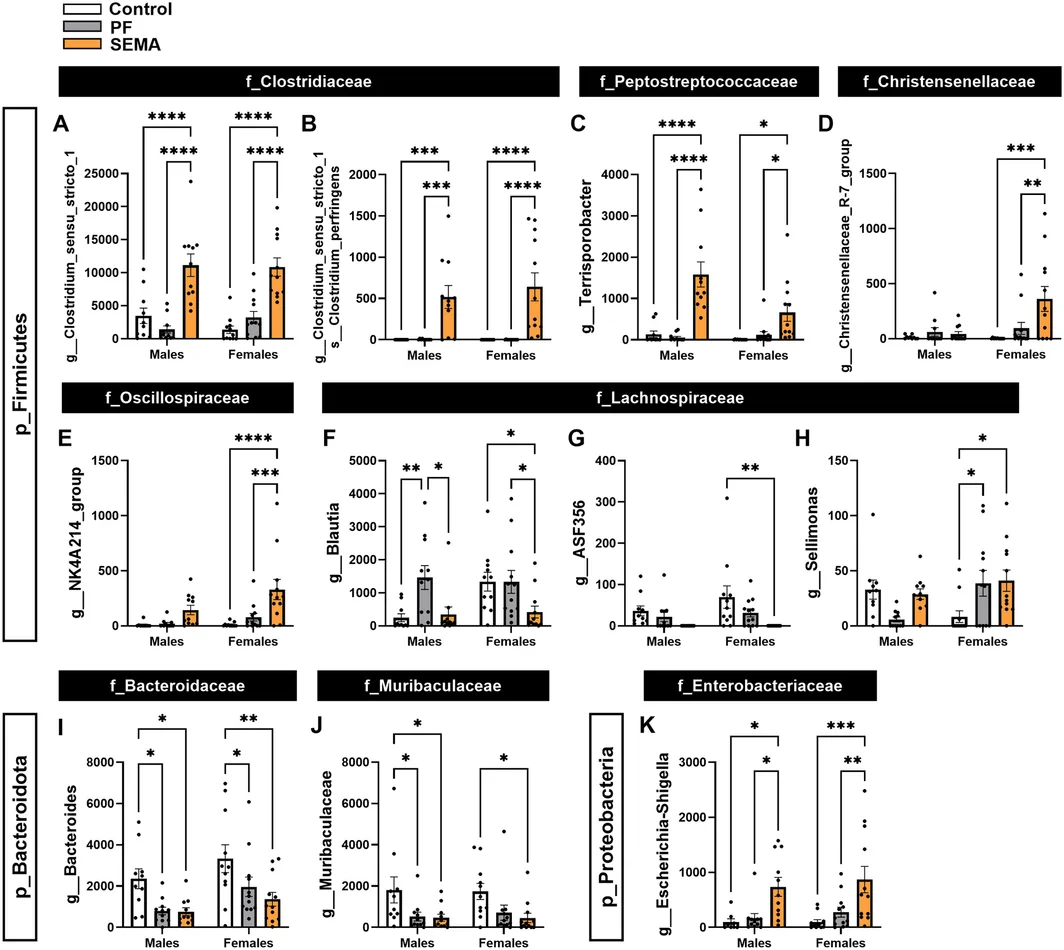
Differential abundance of selected gut microbial taxa across treatment groups. Bar graphs showing taxa counts of bacterial genera in control, pair‐fed (PF), and semaglutide (SEMA)‐treated rats. Representative taxa from the Firmicutes (A–H), Bacteroidota (I–J), and Proteobacteria (K) lineages. Several Firmicutes‐associated taxa were enriched following SEMA treatment, whereas taxa belonging to the Bacteroidota lineage were generally reduced. Data are presented as mean ± SEM. *p < 0.05, **p < 0.01, ***p < 0.001, ****p < 0.0001. *n* = 10–11/group (males); *n* = 11–12/group (females).

Together, these findings indicate that SEMA treatment is associated with enrichment of several Firmicutes taxa and reduction of dominant Bacteroidota‐associated taxa, while some microbial changes appear to be sex‐specific or related to reduced caloric intake.

### 3.6 Differential Taxa Identified by LEfSe

To identify microbial taxa distinguishing treatment groups, we performed LEfSe analysis. A complete list of identified taxa, including LDA scores and *p*‐values, is provided in Table S1.

In males, LEfSe analysis revealed multiple taxa differentially enriched across treatment groups (Figure 5A). The largest number of discriminative taxa were enriched in the SEMA‐treated group (42 taxa), followed by control (35 taxa) and PF rats (19 taxa). Several taxa with the highest LDA scores belonged to the Clostridiaceae family, including *Clostridium sensu stricto 1*, confirming the enrichment of Clostridiaceae‐associated taxa in the SEMA group. Cladogram visualization further illustrated the phylogenetic relationships among these discriminative taxa (Figure 5C), showing that SEMA‐associated taxa were primarily clustered within Firmicutes lineages, whereas control‐associated taxa were more frequently distributed among Bacteroidota taxa, including members of Bacteroidaceae and Muribaculaceae families.

**FIGURE 5:**
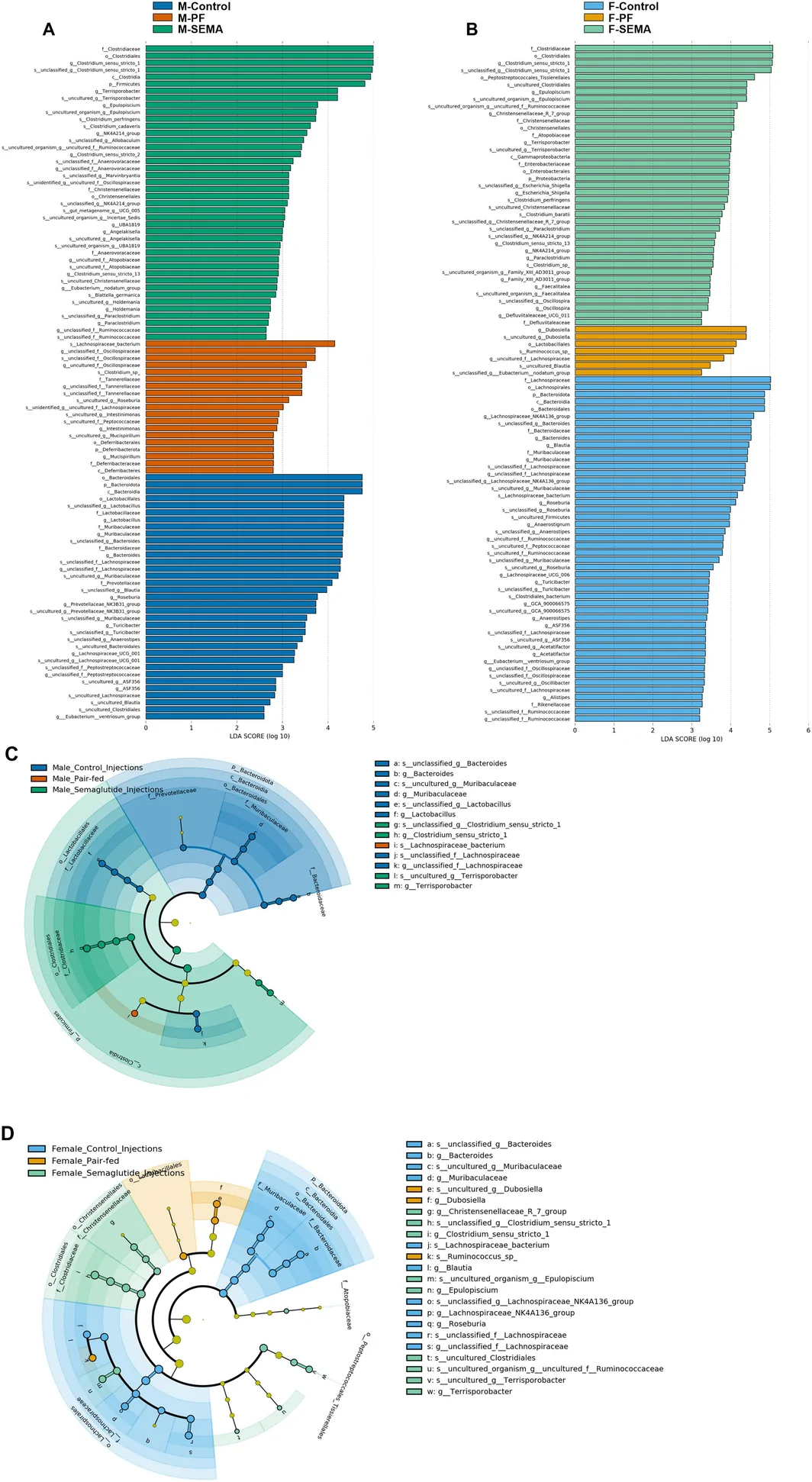
Semaglutide is associated with distinct gut microbial taxa. Linear discriminant analysis effect size (LEfSe) identifying microbial taxa differentially enriched in control, pair‐fed (PF), and semaglutide (SEMA)‐treated rats in males (A) and females (B). Bars represent the LDA score (log10) for each discriminative taxon, indicating the effect size of differential abundance. In males, 42 taxa were enriched in the SEMA group, compared with 35 taxa in control and 19 taxa in PF rats. Several taxa with the highest LDA scores belonged to the Clostridiaceae family, including *Clostridium sensu stricto 1*. In females, 48 taxa were enriched in control, 39 taxa in SEMA, and 7 taxa in PF rats, with several high‐scoring taxa in the SEMA group also belonging to Clostridiaceae‐associated taxa. Colors indicate the treatment group in which each taxon is enriched. Cladograms illustrating the phylogenetic relationships of taxa identified as significantly discriminative by LEfSe in males (C) and females (D). Nodes represent taxonomic levels from phylum to genus, and colored nodes indicate taxa significantly enriched in the corresponding treatment group. Larger node sizes represent higher relative abundance. In males, SEMA‐associated taxa cluster primarily within Firmicutes lineages, whereas control‐associated taxa are more frequently distributed among Bacteroidota taxa, including members of the Bacteroidaceae and Muribaculaceae families. A similar phylogenetic distribution is observed in females. LEfSe analysis was performed using an LDA score threshold of 2.0 and a significance cutoff of *p* < 0.05. *n* = 10–11/group (males); *n* = 11–12/group (females).

In females, LEfSe analysis also identified multiple discriminative taxa across treatment groups (Figure 5B). A total of 48 taxa were enriched in control, 39 taxa in SEMA, and 7 taxa in PF rats. Like in males, several taxa with high LDA scores in SEMA‐treated rats belonged to Firmicutes‐associated families, including Clostridiaceae, Christensenellaceae, and taxa related to *Terrisporobacter*. Cladogram analysis further demonstrated the phylogenetic distribution of these taxa (Figure 5D), highlighting distinct clustering of treatment‐associated microbial lineages and indicating treatment‐specific restructuring of the gut microbial community.

### 3.7 Correlations Between Host Metabolic Parameters and Relative Abundance of Dominant Gut Microbial Phyla

To determine potential associations between host metabolic parameters and gut microbial composition, correlation analyses were performed between physiological measures and the relative abundance of 5 major bacterial phyla.

In males, body weight change was negatively correlated with Firmicutes abundance (*r* = −0.54, *p* = 0.001) and positively correlated with Bacteroidota abundance (*r* = 0.57, *p* = 0.0007) (Figure 6B,C). No significant association was observed between body weight change and Verrucomicrobiota (Figure 6D). Food intake was negatively correlated with Firmicutes (*r* = −0.39, *p* = 0.026) and positively correlated with Proteobacteria abundance (*r* = 0.48, *p* = 0.006) (Figure 6E,F). In addition, the proportion of calories derived from HFD intake was positively correlated with Firmicutes (*r* = 0.54, *p* = 0.002) and negatively correlated with Bacteroidota (*r* = −0.60, *p* = 0.0003) (Figure 6G,H).

**FIGURE 6:**
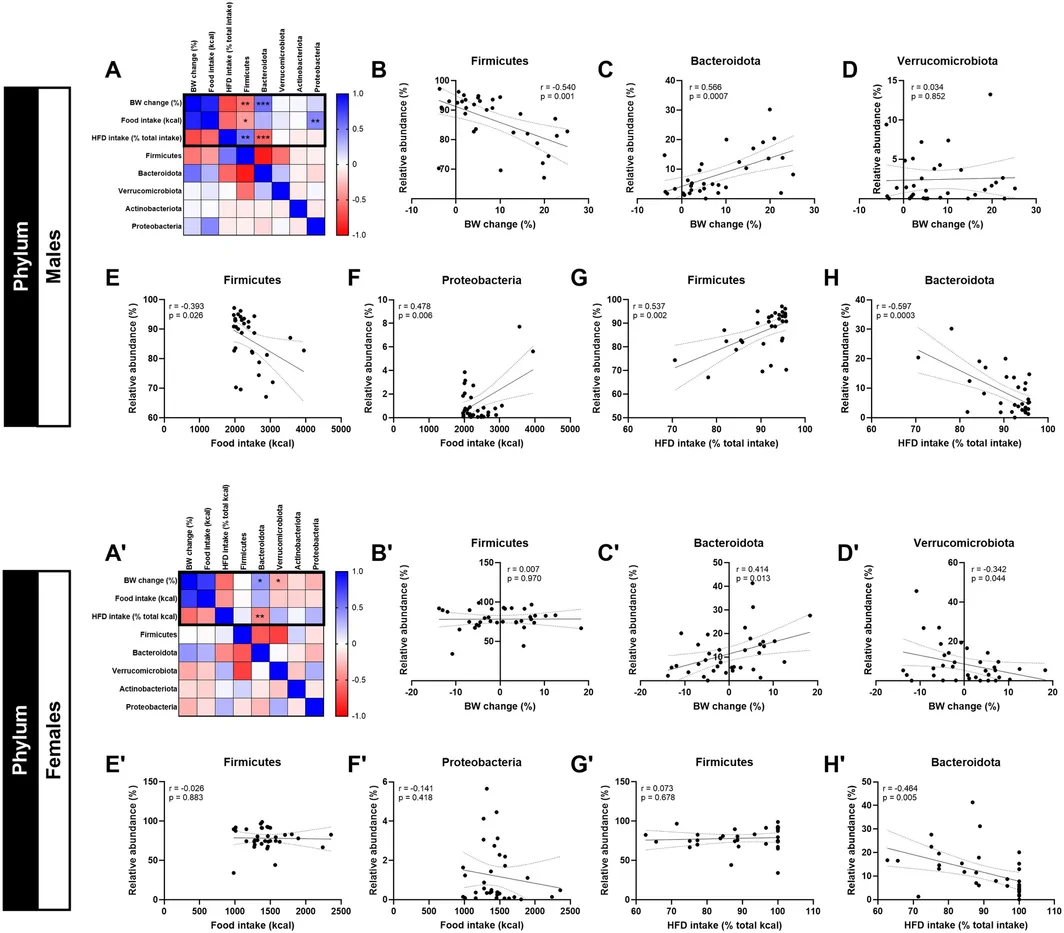
Associations between host metabolic parameters and relative abundance of major gut microbial phyla. Correlation heatmaps showing relationships between host metabolic parameters (body weight change, total caloric intake, and high‐fat diet (HFD) intake expressed as % of total caloric intake) and the relative abundance of five major bacterial phyla in males (A) and females (A'). Color scale represents Pearson correlation coefficients. Asterisks indicate statistically significant correlations between host metabolic parameters and bacterial phyla (*p < 0.05, **p < 0.01, ***p < 0.001). Significance markers are shown only for correlations between host parameters and bacterial phyla. Scatter plots illustrating correlations between microbial phyla abundance and metabolic parameters in males (B–H). Corresponding correlation analyses in females (B′–H′). Each point represents an individual rat, and solid lines indicate linear regression fits. Pearson correlation coefficients (*r*) and *p*‐values are shown in each panel.

In females, fewer significant correlations were observed. Bacteroidota abundance was positively correlated with body weight change (*r* = 0.41, *p* = 0.013) (Figure 6C'), whereas Verrucomicrobiota abundance showed a modest negative correlation with body weight change (*r* = −0.34, *p* = 0.044) (Figure 6D'). Additionally, Bacteroidota abundance was negatively correlated with HFD intake (*r* = −0.46, *p* = 0.005) (Figure 6H'). No significant relationships were detected between Firmicutes abundance and host metabolic parameters in females.

These findings suggest that host metabolic parameters—including body weight change, total caloric intake, and HFD preference—are associated with shifts in the relative abundance of major bacterial phyla, with associations driven by Firmicutes, Bacteroidota, and Proteobacteria in males and only Bacteroidota and Verrucomicrobiota in females.

### 3.8 Correlations Between Host Metabolic Parameters and Relative Abundance of Dominant Gut Microbial Families

To further investigate associations between host metabolic parameters and gut microbial composition at the family level, correlation analyses were performed between physiological measures and the relative abundance of 15 dominant bacterial families.

In males, several bacterial families were significantly associated with host metabolic parameters. Body weight change was negatively correlated with Clostridiaceae (*r* = −0.37, *p* = 0.038) (Figure 7B) and Christensenellaceae (*r* = −0.43, *p* = 0.014) (Figure 7F), while Bacteroidaceae (*r* = 0.47, *p* = 0.006) (Figure 7E) and Muribaculaceae (*r* = 0.45, *p* = 0.01) (Figure 7G) showed a positive correlation with body weight change. No significant relationship was observed between body weight change and Lachnospiraceae (Figure 7C) or Akkermansiaceae (Figure 7D). Food intake was positively correlated with Enterobacteriaceae (*r* = 0.48, *p* = 0.005) (Figure 7J), whereas Lachnospiraceae (Figure 7H) and Muribaculaceae (Figure 7I) did not show a significant association. In addition, the proportion of calories derived from HFD intake was negatively correlated with Bacteroidaceae (*r* = −0.36, *p* = 0.046) (Figure 7L) and Muribaculaceae (*r* = −0.59, *p* = 0.0004) (Figure 7N), while Clostridiaceae (Figure 7K) and Christensenellaceae (Figure 7M) did not show significant relationships.

**FIGURE 7:**
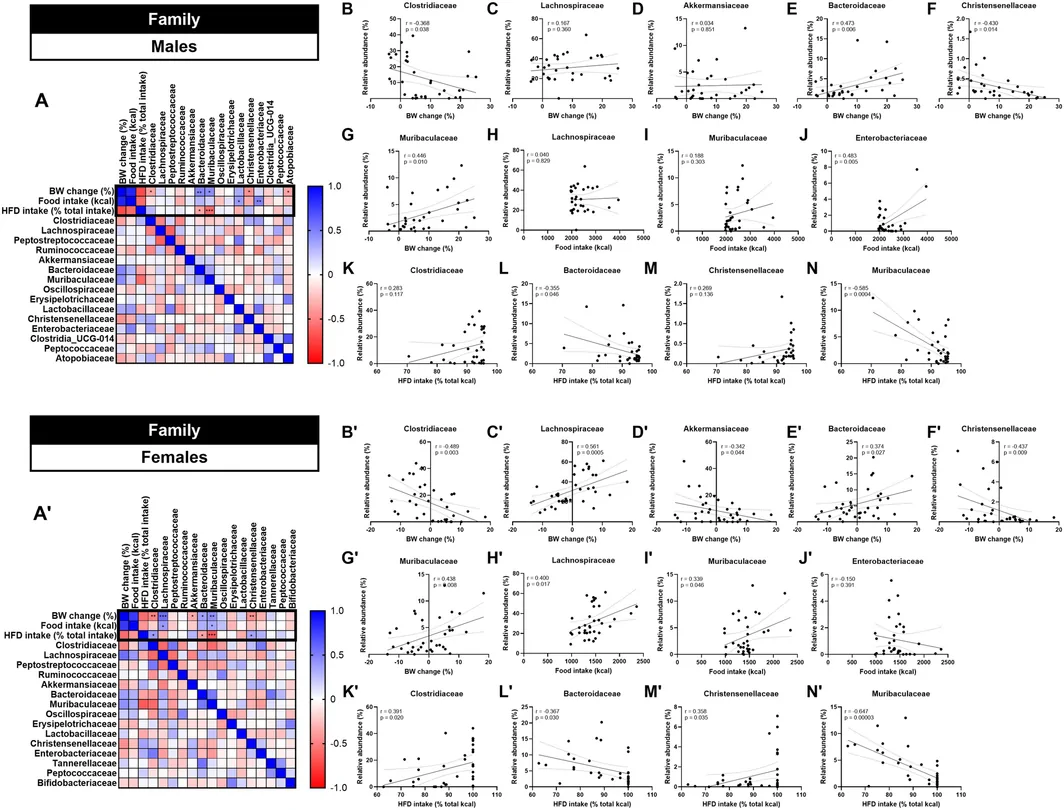
Associations between host metabolic parameters and relative abundance of dominant gut microbial families. Correlation heatmaps showing relationships between host metabolic parameters (body weight change, total caloric intake, and high‐fat diet (HFD) intake expressed as percentage of total caloric intake) and the relative abundance of 15 dominant bacterial families in males (A) and females (A'). Color scale represents Pearson correlation coefficients. Asterisks indicate statistically significant correlations between host metabolic parameters and bacterial families (*p < 0.05; **p < 0.01, ***p < 0.001). Significance markers are shown only for correlations between host parameters and bacterial families. Scatter plots illustrating correlations between bacterial family abundance and metabolic parameters in males (B–N). Corresponding correlation analyses in females (B′–N′). Each point represents an individual rat, and solid lines indicate linear regression fits. Pearson correlation coefficients (*r*) and *p*‐values are shown in each panel.

In females, several correlations were also detected, although the patterns differed from those observed in males. Body weight change was negatively correlated with Clostridiaceae (*r* = −0.49, *p* = 0.003) (Figure 7B'), Akkermansiaceae (*r* = −0.34, *p* = 0.044) (Figure 7D') and Christensenellaceae (*r* = −0.44, *p* = 0.009) (Figure 7F'), whereas Lachnospiraceae (*r* = 0.56, *p* = 0.0005) (Figure 7C'), Bacteroidaceae (*r* = 0.37, *p* = 0.027) (Figure 7E'), and Muribaculaceae (*r* = 0.44, *p* = 0.008) (Figure 7G') showed positive correlations with body weight change. Food intake was positively correlated with Lachnospiraceae (*r* = 0.40, *p* = 0.017) (Figure 7H') and Muribaculaceae (*r* = 0.34, *p* = 0.046) (Figure 7I'). The proportion of calories derived from HFD intake was positively correlated with Clostridiaceae (*r* = 0.39, *p* = 0.02) (Figure 7K') and Christensenellaceae (*r* = 0.36, *p* = 0.035) (Figure 7M'), while Bacteroidaceae (*r* = −0.37, *p* = 0.03) (Figure 7L') and Muribaculaceae (*r* = −0.65, *p* = 0.00003) (Figure 7N') showed a negative correlation with HFD intake.

Overall, these findings suggest that host metabolic parameters—including body weight change, total caloric intake, and HFD preference—are associated with shifts in the relative abundance of specific bacterial families, with sex‐dependent patterns of host–microbiome associations.

### 3.9 Predicted Microbial Functional Pathways

To explore potential functional differences in the gut microbiome across treatment groups, predicted metagenomic pathways were inferred from 16S rRNA gene data using PICRUSt2 and analyzed using LEfSe. A complete list of predicted pathways, including LDA scores and *p*‐values, is provided in Table S2.

In males (Figure 8A), controls showed enrichment of pathways related primarily to carbohydrate and glycan metabolism, including lipopolysaccharide biosynthesis, N‐glycan biosynthesis, the pentose phosphate pathway, and citrate cycle pathways, indicating greater predicted capacity for polysaccharide processing and central carbon metabolism. PF rats displayed enrichment of pathways associated with cellular machinery and regulatory systems, including ribosome and RNA polymerase pathways, as well as flagellar assembly and two‐component signaling systems, suggesting increased predicted transcriptional and motility‐related functions. In contrast, SEMA‐treated rats showed enrichment of pathways involved in amino acid metabolism (e.g., arginine and beta‐alanine metabolism), lipid metabolism (e.g., glycerophospholipid metabolism and secondary bile acid biosynthesis), and xenobiotic degradation pathways, suggesting altered microbial metabolic potential related to nutrient processing and detoxification relative to control and PF groups.

**FIGURE 8:**
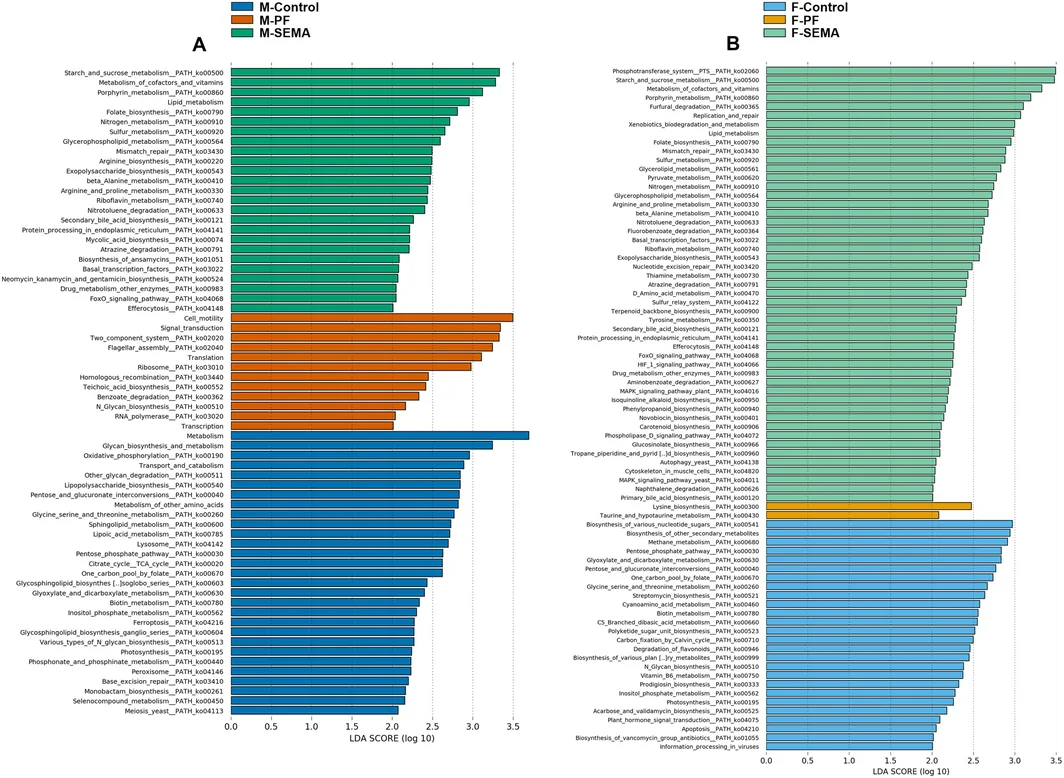
Predicted microbial functional pathways inferred using PICRUSt2. Predicted metagenomic functions were inferred from 16S rRNA gene sequencing data using PICRUSt2 and differential pathway enrichment was assessed using LEfSe analysis (LDA > 2.0, *p* < 0.05). Enriched KEGG pathways are shown for males (A) and females (B). Bars represent the log10 LDA scores indicating the effect size of pathway enrichment. Colors indicate the treatment group in which each pathway is significantly enriched.

In females (Figure 8B), predicted functional pathway analysis revealed broader enrichment associated with SEMA treatment. Control females showed enrichment primarily in carbohydrate metabolism pathways, including the pentose phosphate pathway, nucleotide sugar biosynthesis, and glyoxylate/dicarboxylate metabolism, as well as vitamin and cofactor metabolism pathways. PF females exhibited relatively limited pathway enrichment, mainly related to amino acid metabolism such as lysine biosynthesis and taurine/hypotaurine metabolism. In contrast, SEMA‐treated rats displayed widespread enrichment across multiple metabolic categories, including xenobiotic biodegradation, lipid metabolism, amino acid metabolism, transcription and repair pathways, and diverse secondary metabolite biosynthesis pathways, indicating a broader predicted functional shift in the microbiome following SEMA treatment.

## 4 Discussion

In the present study, we demonstrate that SEMA treatment induces robust and sex‐dependent restructuring of the gut microbiome, which is only partly driven by changes in body weight and caloric intake. Viewed through the lens of interorgan communication, the PF comparison separates two routes by which SEMA reaches the gut microbiome: a brain‐to‐gut route, in which centrally mediated hypophagia and altered diet choice reduce and change the substrates available to the microbiota (changes shared by SEMA and PF rats), and an intake‐independent route (changes unique to SEMA). Across both sexes, SEMA‐treated animals exhibited increased microbial richness but reduced evenness, along with pronounced shifts in overall community composition. These findings indicate that pharmacological weight loss is associated with a reorganization of microbial ecology rather than simply restoring a canonical “healthy” microbial profile upon significant weight loss on SEMA (Lynch and Pedersen 2016; Walter et al. 2020).

SEMA specifically altered alpha diversity in both sexes. An increase in observed richness but reduced evenness by SEMA may indicate that while new microbes have entered the community, the overall structure has become imbalanced, dominated by a few fast‐growing species. This state potentially indicates a specific form of instability often observed in environmental shifts or early dysbiosis, where a few opportunistic taxa outcompete others for resources.

SEMA treatment was characterized by enrichment of Firmicutes‐associated taxa, particularly within the Clostridiaceae family, and reductions in Bacteroidota lineages. Notably, we observed a marked expansion of *Clostridium perfringens* , representing one of the most prominent microbial shifts in the dataset. While early studies proposed that an increased Firmicutes‐to‐Bacteroidota ratio is associated with obesity (Ley et al. 2006), subsequent work has demonstrated that this metric is highly variable and insufficient as a standalone indicator of metabolic health (Sze and Schloss 2016; Indiani et al. 2018; Magne et al. 2020). Our findings further support this revised perspective, suggesting that pharmacological interventions can induce distinct microbial configurations that do not conform to traditional “lean” profiles.

Importantly, several of the taxa altered by SEMA have functional implications for host physiology. The expansion of taxa such as *Clostridium perfringens* and *Escherichia/Shigella*, which possess pathogenic potential under permissive conditions (Kiu and Hall 2018), suggests that drug‐induced microbial restructuring may not uniformly favor beneficial taxa. These changes may arise from altered nutrient availability, shifts in luminal gradients, and modulation of host‐derived factors including bile acids and mucosal signaling, all of which are known to shape microbial community structure (Koh et al. 2016; Cani et al. 2008). The robust reduction in Bacteroidota is somewhat surprising as some studies indicate their abundance persists under caloric restriction (Tanca et al. 2018; Kurup et al. 2021), as they are quick to adapt to depletion in dietary nutrients by utilizing host‐derived nutrient (Fischbach and Sonnenburg 2011). However, there might be a significant difference between fasting while generally in a healthy state of metabolism versus caloric restriction under a model of metabolic disease, or diet‐induced obesity. Bacteroidota are considered to support immune regulation and inhibit pathogens including *C. difficile*; their abundance decreases in inflammatory bowel disease. Thus, it's not unlikely that the reduction induced by caloric restriction and persistent when this restriction is SEMA‐driven does not represent a return to a “healthy” microbiome.

The sex‐specific effect on Akkermansiaceae is also of interest. It's increased by both caloric restriction and SEMA, with the SEMA effect inducing an even more robust expansion compared to equivalent caloric restriction alone. This effect on *Akkermansia* is, however, only present in females; its abundance remains unchanged in males. The changes in abundance are in line with the correlation of *Akkermansia* with weight loss, where greater numbers of Akkermansiaceae are positively associated with greater weight loss, but only in females. This is one example of what could be construed as a positive change in gut microbiome, since Akkermansiaceae are associated with improved gut barrier and improved mucus production in HFD‐fed obesity mice (Everard et al. 2013; Paone et al. 2026). This is thought to reduce systemic inflammation associated with metabolic disease including obesity. Its supplementation was associated with improved metabolic markers in human clinical trials (Depommier et al. 2019; Gofron et al. 2024; Choi et al. 2021).

Another finding unique to females, but also unique to SEMA, and not found under caloric restriction, is the increased numbers of the *Christensenellaceae R‐7* group. They represent a beneficial family of bacteria within the human gut microbiome (within phylum Firmicutes) strongly associated with low body mass index, reduced visceral fat, reduced triglycerides, and overall metabolic health (Mazier et al. 2021; Waters and Ley 2019). They promote healthy metabolism and are typically enriched in healthy individuals rather than those with metabolic disease (Mazier et al. 2021). Thus, this change in microbiome might be considered as positive. This is also supported by the discovered association of higher abundance of Christensenellaceae in rats that lost more body weight during the study. Interestingly, this association was present in both sexes, indicating that this family is associated with positive metabolic changes in both sexes.

Additionally, changes in Lachnospiracea appear to be sex specific. When evaluated on a family level, no change in relative abundance was detected in males, by either caloric restriction or SEMA. Yet in females, SEMA specifically reduced the abundance of Lachnospiraceae by 50%. In females, but not in males, an increased abundance of Lachnospiracea was associated with higher caloric intake and greater body weight gain, supporting a female‐specific interaction with metabolic parameters. The reduction of Lachnospiraceae, key producers of short‐chain fatty acids such as butyrate (Louis and Flint 2017), suggests a shift in microbial metabolic output and substrate utilization. Differences in diet cannot explain the shifts in abundance of this family since divergent effects were observed in the SEMA and PF groups, yet their intake and diet choices were identical. The relationship between the GLP‐1 analogue and Lachnospiraceae becomes even more complex considering that genera in this family can increase intestinal GLP‐1 and gastric inhibitory polypeptide release (Ganamurali and Sabarathinam 2026; Kamath et al. 2026). Abundance of Lachnospiraceae increases in the intestinal lumen of subjects with different diseases, yet it is well documented that the taxa of this family can produce beneficial metabolites for the host (Vacca et al. 2020). *Blautia*, genera within this family, is involved in the control of gut inflammatory processes, atherosclerosis, and maturation of the immune system (Vacca et al. 2020; Zhang et al. 2025; Liu et al. 2021). Increased *Blautia* abundance is considered anti‐inflammatory and is associated with improved outcomes in clinical settings, for example, colorectal cancer, inflammatory pouchitis after ileal pouch–anal anastomosis, and liver cirrhosis (Liu et al. 2021; Holmberg et al. 2024; Chen et al. 2012; Bajaj et al. 2012). In the current study, caloric restriction alone does not alter *Blautia* numbers in females. Yet in SEMA treated females there is a 70% reduction in this genus. In males, SEMA does not change this genus, but caloric restriction alone is associated with a robust increase. Overall, it is difficult to predict whether the changes in Lachnospiracea are positive or negative for health, as in metabolic disease this family along with *Blautia* are associated with impaired metabolic outcomes including diabetes (Vacca et al. 2020; Qin et al. 2012; Kameyama and Itoh 2014).

Consistent with these compositional changes, functional predictions using PICRUSt2 suggest that SEMA‐associated microbial shifts are accompanied by altered metabolic potential, including enrichment of pathways related to lipid metabolism, amino acid metabolism, and xenobiotic processing. These predicted changes may reflect shifts in microbial energy utilization and host–microbe metabolic interactions, potentially mediated through metabolites such as short‐chain fatty acids and bile acid derivatives (Koh et al. 2016; Cani et al. 2008). As these metabolites function as signaling molecules influencing host metabolism and energy homeostasis (Wahlström et al. 2016; Ridlon et al. 2014), such functional shifts may represent an additional mechanism through which SEMA exerts systemic effects. Such microbial signals could complete a gut‐to‐host return path, acting on vagal afferents and enteroendocrine cells to reach brain circuits controlling appetite, and on adipose tissue and liver via the circulation. However, as these predictions are inferred from 16S rRNA data, they should be interpreted cautiously and validated using direct approaches such as metagenomics or metabolomics (Langille et al. 2013; Douglas et al. 2020). In line with this idea, recent studies suggest that at least some metabolic effects associated with SEMA treatment may be transferable through fecal microbiota in male mice (Sun et al. 2025). More generally, microbiome‐dependent regulation of endogenous GLP‐1 signaling further supports the plausibility of bidirectional interactions between GLP‐1 and gut microbial ecology (Kellenberger et al. 2025).

The inclusion of a PF control group provides important insight into the mechanisms underlying these changes. Surprisingly, despite matched caloric intake and diet choice, PF animals exhibited microbial profiles that were clearly distinct from those of SEMA‐treated rats. This indicates that SEMA‐induced microbiome remodeling cannot be attributed solely to caloric restriction but instead reflects additional pharmacological effects. One plausible mechanism involves altered gastrointestinal transit, as GLP‐1RAs are known to slow gastric emptying and influence intestinal motility (Müller et al. 2022; Maselli and Camilleri 2021; Nauck et al. 2021). Reduced transit speed may prolong nutrient exposure in the gut lumen, thereby favoring the expansion of specific microbial taxa and altering fermentation dynamics. Given that intestinal transit time is a key determinant of microbial diversity and metabolic output (Roager et al. 2016; Vandeputte et al. 2016), these effects might represent a central pathway linking GLP‐1 signaling to microbiome restructuring. However, caloric restriction also results in slower GI transit; this is an adaptive response to optimize nutrient absorption capacity (Casirola et al. 1996; Ferraris 1997). This would then suggest that even slower gastric transit may not explain the difference. GLP‐1R are present in the intestinal lumen (Holst et al. 2022), particularly on the epithelium of the colon, and GLP‐1 may act as a local, paracrine system regulating gut functions (Grau‐Bové et al. 2022). Activation of intestinal GLP‐1R is associated with changes in cytokine production and activity of intestinal intraepithelial lymphocytes (Yusta et al. 2015). Moreover, intestinal GLP‐1R activation induces mucin secretion and promotes release of other factors which protect and facilitate healing of the gastrointestinal mucosa (Holst et al. 2022). Increased mucin may favor growth of certain bacterial taxa. Lastly, GLP‐1RA may directly influence gut microbiota by altering intestinal pH, as some studies suggest that GLP‐1 may modulate the intraluminal pH of the small intestine for example (Holst et al. 2022). In addition, because SEMA engages hindbrain and hypothalamic GLP‐1R, altered autonomic outflow to the gut, affecting motility, secretion, and mucosal immune function, could provide a brain‐to‐gut pathway that is independent of food intake (Holst et al. 2022; Bonaz et al. 2018; Huang et al. 2024). All together there are many ways in which GLP‐1R activation may directly affect gut microbiota, in addition to or in opposition to changes induced by the caloric restriction, dietary choice, and weight loss resulting from chronic GLP‐1RA treatment.

Sex‐dependent differences further highlight the complexity of host–microbiome interactions. On a phylum level in males, microbial composition was more tightly correlated with body weight change and dietary parameters. However, females exhibited tighter correlations at the family level. Lachnospiraceae and Akkermansiaceae displayed female‐specific associations, while Enterobacteriaceae were positively correlated with total food intake only in males. These findings are consistent with both clinical and preclinical evidence demonstrating that biological sex influences metabolic responses to GLP‐1RAs (Richard et al. 2016; Lopez‐Ferreras et al. 2023; López‐Ferreras et al. 2019, 2018; Vogel et al. 2016; Rubino et al. 2021), including differences in energy expenditure and thermogenic responses (Byun et al. 2025), and mechanistic work highlighting sex‐dependent differences in GLP‐1 signaling across central and peripheral systems (Börchers and Skibicka 2025). Moreover, sex differences in gut microbiome composition and host–microbe interactions are well established, with both hormonal regulation and sex‐specific physiological factors contributing to distinct microbial profiles (Org et al. 2016; Hases et al. 2023). Factors such as ovarian hormone fluctuations, differences in adiposity, and sex‐specific neural responses to satiety signals may collectively shape the gut environment and contribute to the divergent host–microbiome relationships observed in the present study (Mauvais‐Jarvis 2015).

While the current study clearly shows that SEMA shapes the gut microbiome in a sex dependent manner, future studies are needed to evaluate the trajectory of microbiome remodeling over time and whether early microbial changes precede or follow reduced caloric and body weight. As diet preference was surprisingly altered immediately even by the lowest dose administration, early sampling would not be able to separate the effect of altered preference and microbiome. Similarly, inclusion of chow‐fed control animals would have provided a stronger reference point for interpreting whether SEMA shifted the microbiome toward or away from the pre‐obesogenic state, then the comparisons to past literature discussed here.

Collectively, these findings demonstrate that SEMA induces profound and sex‐dependent restructuring of the gut microbiome that largely cannot be explained by reduced caloric intake alone. Rather than restoring a classical “healthy” microbiome, SEMA appears to establish a distinct microbial configuration associated with negative energy balance, altered nutrient flow, and changes in gastrointestinal physiology. Changes detected were potentially more health‐negative in males, and more mixed in females, with both positive and negative shifts detected. These results underscore the importance of considering the gut microbiome as a dynamic and context‐ and sex‐dependent component of SEMA‐induced weight loss and suggest that microbiome remodeling may represent an integral feature of GLP‐1RA‐mediated metabolic adaptation. In the context of interorgan physiology, they position the gut microbiome as a responsive partner in brain–gut communication during GLP‐1RA treatment, receiving signals both through centrally driven changes in feeding and through intake‐independent drug actions, and potentially signaling back to the brain and peripheral metabolic organs. Most importantly, they also suggest that an effort to balance the microbiome during GLP‐1RA treatment may improve health and potentially ameliorate some of the undesired effects of the treatment. Future studies should determine whether a pre‐ and/or pro‐biotic intervention, aiming to restore a healthy lean microbiome, along with SEMA, leads to better health outcomes.

## Author Contributions

K.P.S. supervised the project. K.P.S. and S.B. designed the experiments. S.B., M.R.S., M.T.B., M.A.K., and M.A. performed the experiments. J.R.W. and C.M. contributed to microbiome sequencing and bioinformatic analysis. S.B. prepared the graphs and figures. S.B. and K.P.S. prepared the initial manuscript draft. M.R.S., M.T.B., M.A.K., M.A., J.R.W., and C.M. revised and approved the manuscript.

## Funding

This work was supported by the National Institute of Diabetes and Digestive and Kidney Diseases (R01DK12932), Canadian Institutes of Health Research, Natural Sciences and Engineering Research Council of Canada.

## Conflicts of Interest

The authors declare no conflicts of interest.

## Supporting information


**Table S1:** Differentially abundant gut microbial taxa identified by LEfSe (linear discriminant analysis effect size) in male and female rats. The ASV table was summarized at the species (L7) taxonomic rank. Sheet 1 (Males) and Sheet 2 (Females) list the biomarker taxa (“Biomarker Lineage,” domain to species) enriched in each treatment group (control injections, semaglutide [SEMA] injections, or pair‐fed [PF]), with the corresponding LDA score (log10) and *p*‐value. Taxa were considered differentially abundant at Kruskal–Wallis *p* < 0.05 and LDA score > 2.0. These data correspond to Figure 5A–D.

**Table S2:** Differentially enriched predicted microbial functional pathways identified by LEfSe analysis of PICRUSt2‐inferred metagenomes in male and female rats. Predicted KEGG orthologs were grouped into KEGG pathways, and pathway abundances were normalized to counts per million (CPM). Sheet 1 (Males) and Sheet 2 (Females) list the pathways (“Enriched Predicted Pathway”: KEGG category, subcategory, pathway name and KO identifier) enriched in each treatment group (control injections, SEMA injections, or PF), with the corresponding LDA score (log10) and *p*‐value. Pathways were considered differentially enriched at Kruskal–Wallis *p* < 0.05 and LDA score > 2.0. These data correspond to Figure 8A–B.

## Data Availability

Data are available upon request.
